# Oxidative Stress Caused by Ozone Exposure Induces Changes in P2X7 Receptors, Neuroinflammation, and Neurodegeneration in the Rat Hippocampus

**DOI:** 10.1155/2021/3790477

**Published:** 2021-11-08

**Authors:** Raúl Velázquez-Pérez, Erika Rodríguez-Martínez, Marlen Valdés-Fuentes, Noemí Gelista-Herrera, Nancy Gómez-Crisóstomo, Selva Rivas-Arancibia

**Affiliations:** ^1^Departamento de Fisiología, Facultad de Medicina, Universidad Nacional Autónoma de México, Ciudad de México, CP 04510, Mexico; ^2^Departamento de Neuropatología, Instituto Nacional de Neurología y Neurocirugía, Ciudad de México, CP 14269, Mexico

## Abstract

Low-ozone doses cause alterations in the oxidation-reduction mechanisms due to the increase in reactive oxygen species, alter cell signaling, and produce deleterious metabolic responses for cells. Adenosine 5′triphosphate (ATP) can act as a mediator in intercellular communication between neurons and glial cells. When there is an increase in extracellular ATP, a modification is promoted in the regulation of inflammation, energy metabolism, by affecting the intracellular signaling pathways that participate in these processes. The objective of this work was to study changes in the P2X7 receptor, and their relationship with the inflammatory response and energy metabolism, in a model of progressive neurodegeneration in the hippocampus of rats chronically exposed to low-ozone doses. Therefore, 72 male rats were exposed to low-ozone doses for different periods of time. After exposure to ozone was finished, rats were processed for immunohistochemical techniques, western blot, quantitative polymerase chain reaction (qPCR), and histological techniques for periodic acid-Schiff staining. The results showed immunoreactivity changes in the amount of the P2X7 protein. There was an increase in phosphorylation for glycogen synthase kinase 3-*β* (GSK3-*β*) as treatment continued. There were also increases in 27 interleukin 1 beta (IL-1 *β*) and interleukin 17 (IL-17) and a decrease in interleukin 10 (IL-10). Furthermore, neuronal glycogen was found at 30 and 60 days, and an increase in caspase 3. An increase in mRNA was also shown for the P2X7 gene at 60 days, and GSK3-*β* at 90 days of exposure. In conclusion, these results suggest that repeated exposure to low-ozone doses, such as those that can occur during highly polluted days, causes a state of oxidative stress, leading to alterations in the P2X7 receptors, which promote changes in the activation of signaling pathways for inflammatory processes and cell death, converging at a progressive neurodegeneration process, as may be happening in Alzheimer's disease.

## 1. Introduction

Repeated exposure to low-ozone doses, like those that can occur during heavily polluted days, is a serious health problem in densely populated cities, due to its association with chronic degenerative diseases [1]. It has been widely demonstrated that chronic exposure to low-ozone doses leads to a deterioration in the response of antioxidant systems, which causes a chronic oxidative stress state. This state is a key factor in the development and evolution of chronic neurodegenerative diseases [2]. Studies in animal models have shown that chronic exposure to low-ozone doses in healthy animals causes a progressive neurodegeneration process that culminates in the intracellular accumulation of beta amyloid 1-42 in the hippocampus [2, 3]. Neurodegenerative diseases such as Alzheimer's occur with a loss of oxide reduction balance, which causes alterations in intracellular signaling pathways, as well as in the transcytosis process, that also affects cell communication, including neurons and glia [4]. Neuronal and glial cells respond to signaling through purinergic receptors [5, 6]. Moreover, mitochondrial function is the main intracellular source of reactive species, since the synthesis of adenosine 5′triphosphate (ATP) is carried out in it, through the electron transport chain (ETC) [7]. In the presence of trauma, ischemia, hypoxia, and inflammation, damaged cells release ATP in large quantities, increasing the extracellular concentrations of this molecule [8]. This leads to an alteration in energy metabolism and inflammatory processes, since neurons, astrocytes, microglia, and endothelial cells have ATP receptors on their membranes, which mediate the processes of neuronal inflammation and death [9–18]. In an oxidoreduction equilibrium, ATP plays a double role, depending on its cellular location. Within the cell, ATP is used as an energy source for the main cellular functions. However, in the extracellular space, it acts on ATP receptors, producing a trophic effect on the development, growth, regeneration, and proliferation of different cell types under normal physiological conditions. It also acts as a mediator of intercellular communication between neurons and glial cells [19–21]. ATP can be found in the synaptic cleft by a process of exocytosis, leading to an increase in extracellular ATP levels [22], which cause activation of purinergic receptors. The activation of receptors to ATP is known to exert its effects by means of the enzyme glycogen synthase kinase 3*β* (GSK3*β*), regulating their activity by mechanisms of phosphorylation; this pathway triggers the inflammatory process activated through the Th1, Th2, and Th17 response, as well as the storage of glycogen in the cell [23, 24]. Furthermore, modulation of the inflammatory response through P2X7 receptors appears to be one of the key pathways in such signaling.

Another effect of the stimulation of ATP receptors is the regulation of energy storage through pathways such as the enzymes GSK3*β* and glycogen synthase (GS). Glycogen is the main glucose energy reserve in the brain and is stored in astrocytic cells and neurons [25, 26]. On the other hand, it has been reported that the increase in the amount of glycogen stored in the neuron is related to cell death. This may explain why glycogen granules are not seen in normal neurons, but they appear in neurons with degenerative damage.

Our team has developed a murine model of progressive neurodegeneration by chronically exposing animals to low-ozone doses similar to what happens on a very polluted day in a city. Our studies have shown that in healthy rats, without any other added factor, exposure to ozone produces the formation of reactive oxygen species, inducing a state of chronic oxidative stress, which causes a process of progressive neurodegeneration and an intracellular increase in beta amyloid 1-42 [2, 3, 27]. Chronic exposure to ozone has also been shown to affect brain repair, because it blocks the neurogenesis process in the hippocampal dentate gyrus [2]. In addition to the above, it has been shown that the oxidative damage caused by chronic exposure to low-ozone doses produces mitochondrial damage and impairs the function of the endoplasmic reticulum, which alters protein synthesis and produces a poor folding of proteins in the hippocampus [28].

The objective of this work was to study changes in the P2X7 receptor, and their relationship with the inflammatory response and energy metabolism, in a model of progressive neurodegeneration in the hippocampus of rats chronically exposed to low-ozone doses.

## 2. Materials and Methods

### 2.1. Experimental Procedures

72 male Wistar rats weighing between 250 and 300 grams were placed in individual acrylic boxes with clean air and ad libitum food conditions (NutriCubo, Purina, Minnetonka, MN, USA). The animals were kept under controlled settings of both temperature and humidity, and the experiments were carried out according to the Official Mexican Standard NOM-062-1999, the National Institutes of Health Guidelines for Animal Treatment and the Ethics Committee of the Faculty of Medicine at the National Autonomous University of México, ethic number FM/DI/043/2019, August 12, 2019.

Animals were randomly divided into 6 experimental groups (*n* = 12). Each group received one of the following treatments: group 1: control, which was treated daily with ozone (O_3_) free air for 4 h; groups 2 to 6 were treated with 0.25 parts per million (ppm) O_3_ for 4 h daily for 7, 15, 30, 60, and 90 days, respectively. At the end of each treatment, 6 animals from each group were deeply anesthetized with 50 mg/kg of weight with sodium pentobarbital and were processed for immunohistochemical and periodic acid-Schiff (PAS) techniques; the remaining 6 were left for western blot and quantitative PCR techniques.

### 2.2. Exposure to Ozone

Exposure to ozone was made according to the method previously reported [2, 27]. Briefly, animals were placed in a hermetically sealed acrylic chamber connected to an ozone generator with a flow of ozone (5 l/s). Ozone concentrations were kept constant for 4 h daily at 0.25 ppm and were monitored throughout the experiment with an ozone monitor (PCI Ozone and Control Systems). The same chamber was used to treat the control group with ozone-free air flow.

### 2.3. Immunohistochemistry

The brains were placed in 30% formaldehyde for 24 h at 4°C and then embedded in paraffin. 5 *μ*m sagittal slices were made from the hippocampus and mounted on slides. The slices were deparaffinized with xylene, rehydrated, passed through a hydration train, and placed in a water bath in an antigen retriever, Diva Decloaker (Biocare Medical, Concord, CA, USA) for 20 min, after which they were placed in a 3% peroxidase blocker for 6 min and passed 3 washes of 10 minutes each with distilled water to remove the solution. For each of the antibodies the P2X7 receptor and GSK3*β* kinase, IL-1*β*, IL-10, IL-17, and caspase 3, the slices were washed three times with tris buffer pH 7.4 and 0.025% Tween 20 and were blocked with 3% bovine serum albumin, and subsequently, they were washed three times with tris buffer pH 7.4 with 0.025% Tween 20 to incubate with the primary anti-rabbit P2X7 antibodies (1 : 100) (Cell Signaling), anti-rabbit pGSK3*β* (S9) (1 : 100) (Cell Signaling), IL-1*β* (1 : 100) (Santa Cruz), IL-10 (1 : 100) (Abcam), IL-17 (1 : 100) (Genetex), and caspase 3 (1: 100) (Biocare). Antibodies were developed using the 3,3-diaminobenzidine DAB Substrate Kit (ScyTek, Logan, UT). The slices were washed with distilled water and counterstained with hematoxylin (ScyTek, Concord, CA). The slices were subsequently passed through a dehydration train and mounted with Entellan®. Results were analyzed using a BX41 Olympus Microscope and photographed using an Evolution-QImagin Digital Camera Kit (Media Cybernetics, Silver Spring, MD, USA).

### 2.4. PAS Staining

The slices were deparaffinized with xylene and rehydrated. The slices were then placed in a periodic acid solution for 5 minutes at room temperature; then, they were washed with distilled water and stained with Schiff reagent for 15 min. Subsequently, they were washed and placed with hematoxylin for one minute, and then, they were washed with distilled water, hydrated, and mounted with Entellan®. The results were analyzed using a BX41 Olympus Microscope and photographed using an Evolution-QImagin Digital Camera Kit (Media Cybernetics, Silver Spring, MD, USA) (Mc, 1948).

### 2.5. Protein Quantification

The hippocampus samples were prepared with 0.017% sodium deoxycholate and 3% trichloroacetic acid; then, they were centrifuged at 5000 g for 30 min at 4°C; the protein content was determined with the Bradford method [29] (Bradford, 1976), and bovine serum albumin was used as the standard curve.

### 2.6. Western Blot

The hippocampal tissue was homogenized and centrifuged; the proteins were mixed with a 6x loading buffer (0.5 M Tris (pH 8.5), 10% SDS, 30% glycerol, 0.1% bromophenol blue, and 100 mM dithiothreitol) and incubated at 100°C for 10 min. Proteins were separated by 12% sodium dodecyl sulfate-polyacrylamide gel electrophoresis technique (SDS PAGE) and transferred to a polyvinylidene fluoride (Immobilon-P Transfer Membranes, Millipore Corporation®, Billerica, MA, USA) membrane. Membranes were blocked with 5% lactose-free milk in 0.1% tris buffer solution with Tween 20 (Tris phosphate buffer/Tween 20 (0.1%)) for 1 h. The membranes were washed three times with Tris-buffered saline with Tween 20 (TBS-T) 0.1% and incubated with goat anti-rabbit IgG and then washed 3 times with TBS-T 0.1%; subsequently, the primary antibodies anti-rabbit P2X7, anti-rabbit (Cell Signaling), pGSK3*β*(S9) (Cell Signaling), IL-1*β* (Santa Cruz), IL-10 (Abcam), IL-17 (Genetex), caspase 3 (Biocare), and anti-rabbit GAPDH (Santa Cruz) and the membranes were incubated for 12 h at 4°C and as a secondary anti-rabbit IgG-coupled horseradish peroxidase (HRP) dilution 1 : 10 000 (anti-rabbit IgG or anti-mouse IgG) diluted 1 : 10,000. The chemiluminescence signal was detected with Immobilon Western Chemiluminescent HRP Substrate® (Millipore Corporation, Billerica, MA, USA).

### 2.7. Obtaining Samples for qPCR

The samples were kept in 1 ml of Trizol® for every 30 mg of hippocampal tissue obtained; afterwards, the samples were homogenized; 200 *μ*l of chloroform was added at room temperature for 3 min and centrifuged at 8000 rpm for 3 minutes. Subsequently, 0.5 ml of isopropanol was added to the aqueous phase and was incubated for 10 minutes at -20°C; they were centrifuged at 10,000 rpm at 4°C for 10 minutes. At this stage, the RNA precipitate forms at the bottom of the tube. The supernatant was discarded, and the pellet was resuspended in 1 ml of 75% ethanol; then, samples were vortexed and then centrifuged for 10 minutes at 8000 rpm at 4°C. Finally, the ethanol was discarded and the RNA was allowed to dry for 20 minutes. The RNA pellet was resuspended in 40 *μ*l of nuclease-free water and stored at -70°C until processing.

### 2.8. qPCR

The quality and quantity of the RNA were estimated spectrophotometrically at 260 and 280 nm, and with a constant amount of RNA, about 2 *μ*g, it was transcribed to cDNA using the SuperScript TM III Reverse transcriptase kit, Oligo (dT) 12-18 Primer, RNaseOUT ™ recombinant ribonuclease inhibitor, and deoxynucleotide triphosphates (dNTP). Set polymerase chain reaction (PCR) grade (Invitrogen, CA, USA). Amplification was performed in triplicate on a MIC® real-time PCR detection system. The oligonucleotide sequences used are shown in Table 1. Amplification was carried out with the Luna Universal qPCR® master mix, in a final volume of 25 *μ*l; the final reaction contained cDNA (1/100) and 0.5 *μ*M of each of the primer pairs, according to the following protocol: activation of Taq DNA polymerase and DNA denaturation at 95°C for 60 s, followed by 40 cycles of amplifications from 15 s to 95°C and 30 s to 60°C. The results obtained were analyzed by the 2-*ΔΔ*CT method, and the cycle threshold was normalized with the constitutive gene used, the Rps 18 gene, to calculate the mRNA levels of the genes.

### 2.9. Statistical Analysis

According to the data distribution, the western blot and qPCR results were analyzed using a nonparametric Kruskal-Wallis test followed by the Mann-Whitney *U* tests. The number of cells is expressed as the mean number of neurons per field for each treatment; the level of significance in this study is ^∗^*p* < 0.05.

Materials and Methods should contain sufficient detail so that all procedures can be repeated. It may be divided into headed subsections if several methods are described.

## 3. Results and Discussion

### 3.1. Results

#### 3.1.1. Effect of Chronic Exposure to Low-Ozone Doses on P2X7 Receptors

**Figure 1:**
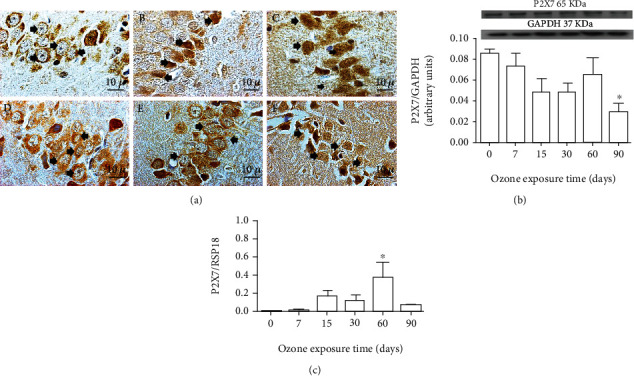
Effect of chronic exposure to low doses of ozone on P2X7 receptors. (a) Effect of ozone exposure on P2X7 receptor immunoreactivity. The photomicrographs are representative of the changes in immunoreactivity against the P2X7 receptor in the CA3 region of the hippocampus for the following treatments: (A) ozone-free air; (B) 7 days of ozone; (C) 15 days of ozone; (D) 30 days of ozone; (E) 60 days of ozone; (F) 90 days of ozone. Arrows show P2X7 immunoreactive neurons. Calibration bar = 10 *μ*m (100x). (b) Effect of ozone treatments on P2X7 proteins. The results showed a decrease in the expression of the P2X7 protein after 90 days of exposure to ozone (^∗^*p* < 0.05). (c) Effect of ozone exposure on P2X7 receptor mRNA. There is a significant increase after 60 days of ozone treatment (^∗^*p* < 0.05).

**Figure 2:**
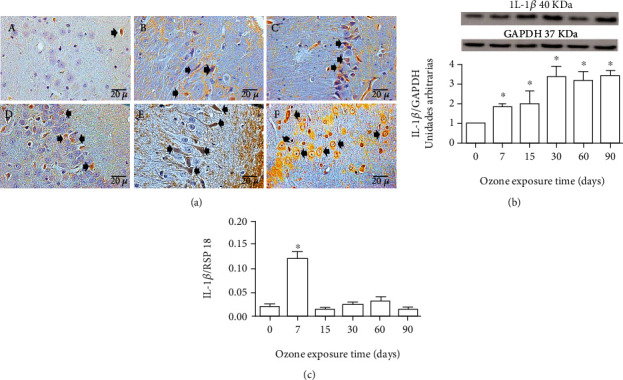
Effect of exposure to low doses of ozone on the expression of IL-1*β* in CA3 of the hippocampus. (a) Photomicrographs are representative of IL-1*β* immunoreactivity in hippocampus CA3: (A) ozone-free air; (B) 7 days of ozone; (C) 15 days of ozone; (D) 30 days of ozone; (E) 60 days of ozone; (F) 90 days of ozone. Note an increase in IL-1*β* immunoreactivity at 15, 30, 60, and 90 days of ozone exposure. Arrows show IL-1*β* immunoreactive neurons. Calibration bar = 20 *μ*m (40x). (b) Effects of ozone treatment on IL-1*β* protein. There is an increase in IL-1*β* protein levels after ozone exposure at 7, 15, 30, 60, and 90 days compared to control groups (^∗^*p* < 0.05). (c) Effects of ozone treatment on IL-1*β* mRNA levels. The results show an increase in the expression of mRNA in response to the exposure of 7 days to ozone (^∗^*p* < 0.05).

Our results showed a modification in morphology as ozone exposure increases (Figure 1), as well as changes in immunoreactivity for the P2X7 receptor in the CA3 region of the hippocampus of rats. We can also observe in Figure 1(a) a change in the immunoreactivity of the P2X7 receptor from the cytoplasm to the nucleus, depending on the time of exposure to ozone (30 and 60 days). The immunoreactivity to P2X7 was distributed mainly in the cytoplasm and cell membrane. At 7, 15, and 90 days, immunoreactivity to P2X7 receptors was observed both in the nucleus and in the cytoplasm (Figure 1(a)) Western blot analysis for P2X7 receptors showed a significant decrease in proteins at 90 days of exposure to ozone (^∗^*p* < 0.05) (Figure 1(b)) compared to the control group. Regarding the changes in the messenger levels of the P2X7 receptor, an increase can be observed after 60 days of exposure to ozone, when compared with the control group (Figure 2(c)).

### 3.2. Effect of Exposure to Low-Ozone Doses on Phosphorylation of Glycogen Synthase Kinase 3*β* (GSK3*β*)

**Figure 3:**
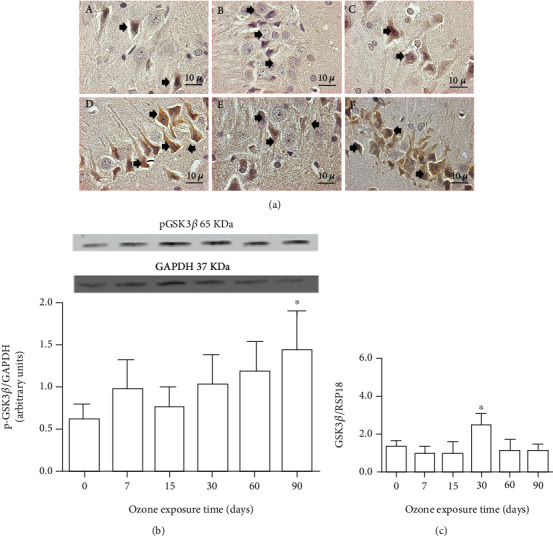
Effect of exposure to low doses of ozone on the kinase p-GSK3*β*. (a) Effect of ozone exposure on immunoreactivity of p-GSK3*β* kinase. Photomicrographs are representative of immunoreactivity changes against p-GSK3*β* kinase in the CA3 region of the hippocampus for the following treatments: (A) ozone-free air; (B) 7 days of ozone; (C) 15 days of ozone; (D) 30 days of ozone; (E) 60 days of ozone; (F) 90 days of ozone. There is an increase in immunoreactivity against the kinase p-GSK3*β*, after 15, 30, and 90 days of treatment with ozone. Arrows show neurons immunoreactive for p-GSK3*β*. Calibration bar = 10 *μ*m (100x). (b) Effect of ozone treatments on GSK3*β* protein. The results showed an increase in the expression of phosphorylated GSK3*β* kinase at 90 days of exposure (^∗^*p* < 0.05). (c) Effects of ozone treatment on GSK3*β* mRNA levels throughout the hippocampus. Note a significant difference at 30 days of ozone exposure with respect to the control group (^∗^*p* < 0.05).

Exposure to low-ozone doses promotes phosphorylation of glycogen 3*β* synthase kinase; this phosphorylation is affected according to the time of exposure of ozone. Photomicrographs showed increased immunoreactivity of phosphorylated GSK*β* kinase at 30 and 90 days of ozone exposure regarding controls (Figure 3(a)). Western blot analysis for phosphorylated GSK3*β* kinase showed a significant increase at 90 days of ozone exposure when compared to the control group (^∗^*p* < 0.05) (Figure 3(b)). Regarding the changes in the messenger levels of GSK3*β*, an increase can be observed at 30 days of exposure to ozone with respect to the control group. (Figure 3(c)).

### 3.3. Effect of Ozone Exposure on IL-1*β*

Exposure to low-ozone doses caused increased immunoreactivity to IL-1*β* according to the time during which the groups were exposed to ozone. Photomicrographs showed increased immunoreactivity of IL-1*β* from 7 days until the end of the treatment with respect to the control group (Figure 2(a)). Western blot analysis for IL-1*β* also showed a protein increase after 7 days and until 90 days of ozone exposure (^∗^*p* < 0.05) (Figure 2(b)). Regarding the changes in the messenger levels of IL-1*β*, an increase at 7 days can be observed when compared with the control group (Figure 2(c)).

### 3.4. Effect of Ozone Exposure on IL-10

**Figure 4:**
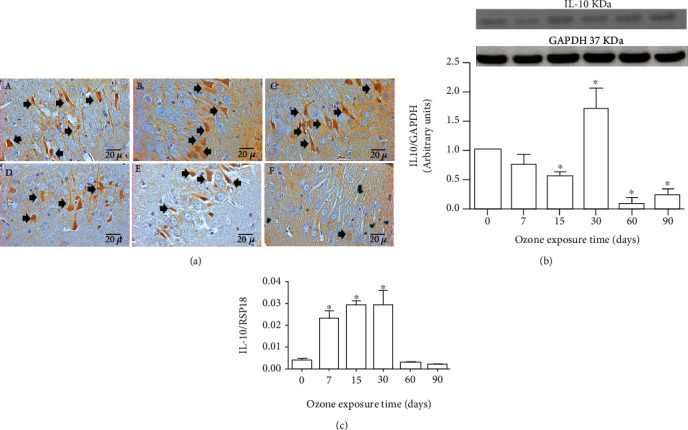
Effect of low-dose ozone on IL-10 expression in the hippocampus CA3. (a) Photomicrographs are representative of changes in immunoreactivity against IL-10: (A) ozone-free air; (B) 7 days of ozone; (C) 15 days of ozone; (D) 30 days of ozone; (E) 60 days of ozone; (F) 90 days of ozone exposure. The control group showed high immunoreactivity for IL-10, while we observed a decrease in it at 60 and 90 days of exposure to ozone. Arrows show IL-10 immunoreactive neurons. Calibration bar = 20 *μ*m (40x). (b) Effects of ozone treatment on IL-10 protein. Note that the IL-10 protein decreases its levels at 30, 60, and 90 days compared to the control groups (^∗^*p* < 0.05). (c) Effects of ozone treatment on IL-10 mRNA levels. The results show an increase in mRNA expression at 7, 15, and 30 days of exposure to ozone (^∗^*p* < 0.05).

Exposure to low-ozone doses caused IL-10 immunoreactivity to decrease according to the time during which the groups were exposed to ozone. Photomicrographs showed an immunoreactivity decrease of IL-10 exhibited at 30, 60, and 90 days of treatment to ozone exposure (Figure 4(a)). Western blot analysis for IL-10 also showed a significant increase at 30 days and a decrease at 15, 60, and 90 days of ozone exposure (^∗^*p* < 0.05) (Figure 4(b)). Regarding the changes in the messenger levels of IL-10, there was an increase at 7, 15, and 30 days followed by a decrease at 60 and 90 days of ozone exposure compared to the control group (Figure 4(c)).

### 3.5. Effect of Ozone Exposure on IL-17

**Figure 5:**
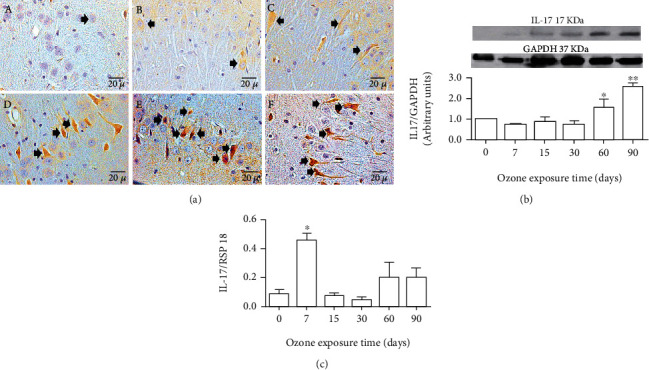
Effect of exposure to low doses of ozone on IL-17 expression in hippocampal CA3. (a) Photomicrographs are representative of changes in immunoreactivity against IL-17: (A) ozone-free air; (B) 7 days of ozone; (C) 15 days of ozone; (D) 30 days of ozone; (E) 60 days of ozone; (F) 90 days of ozone. The control group showed low immunoreactivity. However, we observed an increase in immunoreactivity for IL-17 at 60 and 90 days of exposure to ozone. Arrows show IL-17 immunoreactive neurons. (b) Effects of ozone treatment on IL-17 protein. The result shows increased rat hippocampal CA3 IL-17 protein levels after ozone exposure at 60 and 90 days compared to control groups (^∗^*p* < 0.05). (c) Effects of ozone treatment on IL-117 mRNA levels. Note an increase in mRNA expression after 7 days of exposure to ozone (^∗^*p* < 0.05).

Exposure to low-ozone doses caused increased immunoreactivity to IL-17 with respect to ozone exposure time (Figure 5). Photomicrographs showed that IL-17 immunoreactivity gradually increased as ozone exposure time progressed from 7 days until the end of the treatment to ozone (Figure 5(a)). Western blot analysis for IL-17 also showed a significant protein increase at 60 and 90 days of ozone exposure (^∗^*p* < 0.05) (Figure 5(b)). Regarding the changes in the messenger levels of IL-17, a significant increase at 7 days can be observed after ozone exposure (Figure 5(c)).

### 3.6. Effect of Ozone Exposure on Neuronal Glycogen

**Figure 6:**
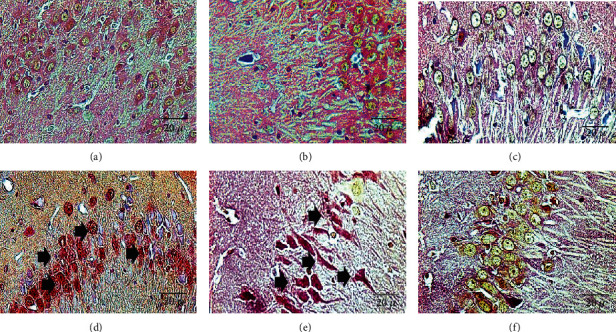
Effect of exposure to low doses of ozone on glycogen storage by PAS staining. Representative micrographs of glycogen staining in the CA3 region of the hippocampus: (a) ozone-free air; (b) 7 days of ozone; (c) 15 days of ozone; (d) 30 days of ozone; (e) 60 days of ozone; (f) 90 days of ozone. The micrographs show an increase in glycogen storage at 30 and 60 days of ozone exposure compared to the control group. The arrows show the neurons that contain glycogen. Calibration bar = 20 *μ*m (40x).

Effect of exposure to low-ozone doses on glycogen storage (Figure 6). Chronic exposure to ozone modified glycogen storage, and this storage increased according to the exposure time, where an increase in glycogen was shown from 30 to 60 days (Figure 6(a)). The number of cells that show glycogen staining also had a significant increase at 30 and 60 days of exposure to ozone in regard to the control (^∗^*p* < 0.05) (Figure 6(b)).

### 3.7. Effect of Ozone Exposure on Caspase 3

**Figure 7:**
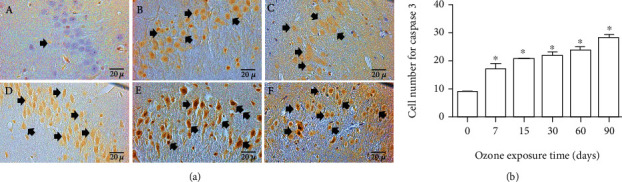
Effect of exposure to low doses of ozone on the expression of caspase 3 in hippocampal CA3. (a) Photomicrographs are representative of changes in caspase 3 immunoreactivity: (A) ozone-free air; (B) 7 days of ozone; (C) 15 days of ozone; (D) 30 days of ozone; (E) 60 days of ozone; (F) 90 days of ozone. Arrows show nuclei of caspase 3 immunoreactive neurons. Calibration bar = 20 *μ*m (40x). The control group showed low immunoreactivity. However, we observed an increase in caspase 3 immunoreactivity in the nucleus of hippocampal CA3 neurons at 60 and 90 days of exposure to this gas. (b) Effect of exposure to low doses of ozone on the number of neurons immunoreactive to caspase 3. Note the significant increase in the number of neurons showing immunoreactivity in their nucleus at 7, 15, 30, 60, and 90 days of treatment (^∗^*p* < 0.05).

Exposure to low-ozone doses caused increased immunoreactivity to caspase 3 which was correlated with ozone exposure time (Figure 7). Photomicrographs showed that caspase 3 immunoreactivity gradually increased as ozone exposure time progressed from 7 days until the end of the treatment (Figure 7(a)). The number of cells that presented immunoreactivity to caspase 3 also showed an increase from 7 days until 90 days of ozone exposure in regard to the control group (^∗^*p* < 0.05) (Figure 6(b)).

## 4. Discussion

As has been widely demonstrated, exposure to low doses of ozone induces a neurodegenerative process in the hippocampus, depending on the time of exposure to this gas [27]. On the other hand, the oxidative stress state that occurs secondary to ozone exposure causes the alteration of the signaling pathways that participate in inflammatory processes and cell death, in addition to inducing neurodegeneration in the hippocampus [30].

This work shows that the immunoreactivity of the P2X7 receptor presents an intracellular modification in neurons of the CA3 region of the hippocampus. These changes in the location of the immunoreactivity of the cytoplasm to the nucleus as a function of the time of exposure to ozone are also accompanied by alterations in cell morphology (Figure 1(a)). The results obtained from all the hippocampal tissue did not present significant protein levels until 60 days. However, we can observe a significant increase in mRNA at 60 days, as well as a significant decrease in both protein and mRNA for P2X7 at 90 days; this decrease coincides with the experimental group with the highest amount of neuronal death. These results indicate that the chronic oxidative stress state causes alterations in the expression and distribution of the P2X7 receptor, which is related to the advancement of the neurodegenerative process (Figure 7); the observed morphological changes were compatible with a progressive process of cell death in the pyramidal neurons of CA3 in the hippocampus. The neurodegenerative effect caused by exposure to low doses of ozone in healthy rats has also been previously shown by our work group [3, 27, 30].

During a state of chronic oxidative stress, as the results of this work show, changes occur in ATP receptors, since oxidative stress induces the entry of calcium into the cell, as well as leading to mitochondrial alteration [28, 31] and to an increase in phosphorylation pathways [2, 28]. Changes in the intracellular P2X7 receptor distribution result in the alteration of the immunoreactivity of the enzyme p-GSK3*β* (Figure 3(a)). This enzyme is negatively regulated by phosphorylation in serine 9 and 21 residues located in its N-terminal, decreasing its catalytic activity [32].

These modifications generate a structural change in the enzyme, generating a pseudosubstrate that does not have access to its catalytic site [33]. Taking into account the above, in this work, the antibody was used to show the changes in the GSK3*β* immunoreactivity of the enzyme marked phosphorylation in serine 9 (Figures 3(a) and 3(b)).

The results in this experiment show that the changes observed in the P2X7 receptors led to the phosphorylation of GSK3*β*. The inhibition of GSK3*β* by phosphorylation and of the enzymes found downstream in the signaling cascade modifies its activity [34, 35], as shown in this work in Figures 3(a) and 3(b).

In previous work, we have shown that the oxidative stress state produces mitochondrial and endoplasmic reticulum damage, causing protein misfolding in hippocampal cells [28, 31, 36]. In addition to the above, the damage to the mitochondria, which accompanies the process of progressive neurodegeneration, promotes a decrease in the levels of available ATP in the cells, thus modifying the cellular energy metabolism [26], losing the regulation of membrane functions and altering the internal environment of cells due to the failure of ATP-dependent pumps [37]. Therefore, oxidative stress caused by ozone exposure causes mitochondrial damage, leading to a loss of regulation of intracellular energy, which produces an influx of calcium, increasing cellular damage and ATP release by exocytosis, thus increasing extracellular ATP, which acts on purinergic membrane receptors, leading to alteration of intracellular signaling pathways that depend on of these receptors [38]. Therefore, these changes may be reflected in alterations in the regulation of the canonical pathways of cell survival, also altering the signaling cascades which are involved in cellular energy metabolism [26], and in the inflammation pathways [39, 40], as can be seen in Figures 2–5.

On the other hand, it has been reported that the activation of the purinergic P2X7 receptors causes an increase in the inflammatory response [40]. The stimulation of these purinergic receptors can act directly on the formation of the inflammasome, or through the nuclear factor kappa-light-chain-enhancer of activated B cell (NF*κ*B) [41, 42], giving rise to the release of proinflammatory interleukins such as IL-1*β* (Figure 2). GSK3 also modulates the expression of cytokines with inflammatory activity [43]. Therefore, the loss of modulation of the inflammatory response may be occurring because of P2X7 but also due to GSK3*β* and other stimuli produced directly by oxidative stress, such as its effect on the activation of membrane kinases, which also leads to the activation of the NF*κ*B. Finally, an inflammatory response type Th1, Th2, and Th17 is activated, as shown in the results (Figures 2–5); we can also observe an increase in IL-17 (Figure 5(a)) in CA3 neurons of the hippocampus of exposed rats at low-ozone doses. Furthermore, in Figure 4, the results indicate a decrease in IL-10, which coincides with the onset of high neuronal death in our model [2], as can be seen in Figure 7(a), where there is an increase in immunoreactivity to caspase 3.

The results show that there is a significant increase in IL-1*β* immunoreactivity and in the amount of IL-1*β* protein from 7 days to the end of treatment, as indicated in Figure 2. These results show that low-dose exposure to ozone causes an increase in the inflammatory response type Th1, which increases as the time of exposure to ozone passes (Figure 2), and that is present during the process of progressive neurodegeneration. Therefore, this may indicate that it could be one of the factors whose increase maintains the process of cell death, leading to the progression of the neurodegenerative disease over time. The decrease in IL-10 in the CA3 hippocampus, which is observed in Figure 4, occurs when in this model used, the neuronal death process becomes irreversible after 30 days of exposure to ozone, and the inflammatory response has lost its regulation. This decrease in IL-10 is a key factor that prevents the inflammatory response from self-limiting. This interleukin, which is part of the Th2-type response, under normal circumstances plays a key role in the regulation and self-limitation of the inflammatory response [44].

In addition, these alterations in IL-1*β* and IL-10 coincide temporarily with an increase in IL-17; the increase of this interleukin, which is part of the Th17 response, is related to the maintenance of a persistent and irreversible chronic inflammation [45] process which is present in many degenerative diseases.

Moreover, since the central nervous system has the highest glucose consumption with respect to size and weight of the entire organism [46], this consumption is highly regulated, and considering glycogen is the main energy supply of neurons, an alteration in the metabolic processes of glucose plays a crucial role in neural survival and in the development of neurodegenerative diseases. Therefore, the stimulation of the P2X7 receptors, by producing the phosphorylation of GSK3*β* in serine 9, can cause the accumulation of neuronal glycogen (Figure 6) at 30 and 60 days through the alteration in glutamyl synthase enzyme [33].

However, during a state of oxidative stress caused by ozone, an increase in the amount of stored glycogen occurs in the neurons. These changes could be directly related to deficits in energy metabolism, such as intracellular ATP deficiency, as well as the changes of the enzymes that participate in the glycogen synthesis or in the metabolic pathway due to alteration in the P2X7 receptors. This causes inhibition of GSK3*β*, which promotes a decrease in the disposition of glucose 6 phosphates, and alterations in the allosteric regulation between glucose and glycogen-binding enzymes. Therefore, glycogen deficiency caused by an oxidative stress state can be a crucial factor that induces neuronal death and the advancement of the neurodegenerative process in the brain. The GSK3*β* enzyme is key in intracellular signaling, since the activation and inactivation of a series of pathways related to the inflammatory response and the metabolic response of the cell, such as glycogen disposition, depend on its change in phosphorylation in the neuron, as shown in the results of this work (Figure 1). Therefore, when GSK3*β* is inhibited, the regulation of glycogen storage in the neuron is lost (Figure 1). As a result, the availability of energy in the neuron, analyzed with the PAS technique to observe the storage of glycogen [47], shows a significant increase in the amount of glycogen from 30 days of ozone exposure to 90 days of treatment, as seen in Figures 1(a) and 1(b). This increase occurs throughout the ozone treatment, reaching a maximum at 90 days. This means that the accumulation of glycogen in the brain during the process of progressive neurodegeneration could represent a failed attempt by neurons to survive during oxidative stress and the energy deficit caused by it [48, 49]. All these alterations in P2X7 receptors, interleukins, and glycogen coincide when, in this model, the neurodegenerative process becomes irreversible, due to neuronal death as shown in Figure 7.

Finally, these results indicate that oxidative stress caused by exposure to low-ozone doses produces an alteration in the signaling of the P2X7 receptors and that this change in the signaling of these receptors causes an inhibition by phosphorylation of the GSK3*β* enzyme, which produces the alteration of GS that contributes to the accumulation of glycogen in the neurons, since the enzyme of GSk3*β* has an important role in regulating the neuron's ability to store glycogen. Finally, in a loss of redox balance, extracellular ATP levels increase due to cellular release, which causes loss of regulation in the inflammatory response and alteration of energy metabolism, leading to the death of neurons (Figure 7) during the advancement of the neurodegenerative process.

## 5. Conclusions

These results allow us to conclude the following: (1) repeated exposure to low doses of ozone causes alterations of the P2X7 receptors; (2) changes in the P2X7 receptors inhibit the GSK3*β* enzyme by phosphorylation; (3) GSK3*β* is a key factor that, when inhibited together, produces metabolic alteration and loss of regulation in the immune response; (4) alterations in the ATP and GSK3*β* receptor cause a decrease in glycogen storage by neurons, which contributes to neuronal damage and death; (5) the convergence of these factors leads to a neurodegenerative process and its maintenance over time. Therefore, all these alterations caused by ozone are a key factor in the process of progressive neurodegeneration, similar to what occurs in Alzheimer's disease.

## Figures and Tables

**Table 1:** Oligonucleotide sequence used for the evaluated genes.

	Sense	Antisense
GSK3*β*	ACC GTA TCT CCT GAG TTC CA T	GTC CAG CTT GAC CAC AGT TTA T
P2X7	CTC CAT GAG CTT TGT ACA AGG	TGC TGA TGT ACC AGT TGG GG
IL-1*β*	CTC CAT GAG CTT TGT ACA AGG	TGC TGA TGT ACC AGT TGG GG
IL-10	AAG GCC ATG AAT GAG TTT GAC AT	CGG GTG GTT CAA TTT TTC ATT T
IL-17	ACT TTC CGG GTG GAG AAG AT	CTT AGG GGC TAG CCT CAG GT
Ribosomal protein S18 (Rps 18)	TTC AGC ACA TCC TGC GAG TA	TTG GTG AGG TCA ATG TCT GC

## Data Availability

The data used to support the findings of this study are available from the corresponding author on reasonable request.
